# Effects of rapid thermal annealing on the optical properties of strain-free quantum ring solar cells

**DOI:** 10.1186/1556-276X-8-5

**Published:** 2013-01-02

**Authors:** Jiang Wu, Zhiming M Wang, Vitaliy G Dorogan, Shibin Li, Jihoon Lee, Yuriy I Mazur, Eun Soo Kim, Gregory J Salamo

**Affiliations:** 1 State Key Laboratory of Electronic Thin Films and Integrated Devices, University of Electronic Science and Technology of China, Chengdu, 610054, People's Republic of China; 2 Institute for Nanoscience and Engineering, University of Arkansas, Fayetteville, AR, 72701, USA; 3 College of Electronics and Information, Kwangwoon University, Nowon-gu, Seoul, 139-701, South Korea

**Keywords:** Photoluminescence, Photovoltaic cells, Quantum rings, Rapid thermal annealing, Droplet epitaxy

## Abstract

Strain-free GaAs/Al_0.33_Ga_0.67_As quantum rings are fabricated by droplet epitaxy. Both photoresponse and photoluminescence spectra confirm optical transitions in quantum rings, suggesting that droplet epitaxial nanomaterials are applicable to intermediate band solar cells. The effects of post-growth annealing on the quantum ring solar cells are investigated, and the optical properties of the solar cells with and without thermal treatment are characterized by photoluminescence technique. Rapid thermal annealing treatment has resulted in the significant improvement of material quality, which can be served as a standard process for quantum structure solar cells grown by droplet epitaxy.

## Background

Since the proposal of intermediate band concept for high-efficiency solar cell, great efforts have been devoted to intermediate band solar cells (IBSCs). Luque and Martí have theoretically predicted that a single-junction solar cell with an intermediate band can be used to assist multiple spectral band absorption and to obtain ultrahigh efficiency up to 63% [1]. Several approaches have been taken to achieve IBSCs, such as quantum dots (QDs) and impurity bands [2]. Among these approaches, most of the current studies on IBSCs have been focused on QDs, and prototype QDIBSCs have been demonstrated [3,4]. The discrete energy levels of electrons in the QDs form energy bands which can serve as intermediate bands. However, the intermediate band impact on the cell performance is still marginal, mainly due to the high recombination rate in strongly confined QDs and low absorption volume of QDs.

Sablon et al. have demonstrated that QDs with built-in charge can suppress the fast recombination and thus prompt electron intersubband transitions in QDs [5]. On the other hand, several groups reported that strain-compensated QDs can be used to increase the number of QD layers and thus the overall absorption volume [6,7]. Recently, strain-free nanostructures grown by droplet epitaxy have been proposed and demonstrated for photovoltaic applications [8,9]. Moreover, strain-free nanostructures have also gained popularity in other optoelectronic devices, such as lasers and photodetectors [10,11]. In order to better understand the optical properties of these unique nanostructures and to fabricate high-performance optoelectronic devices, it is critical to gain further insight into the optical properties of droplet epitaxial strain-free nanostructures.

In this letter, strain-free quantum ring solar cells were fabricated by droplet epitaxy. Rapid thermal annealing (RTA) is used to improve the optical quality of the solar cells. The optical properties of the quantum ring solar cells before and after RTA treatment are studied. The post-growth annealing of epitaxial nanostructures is considered to be important in optoelectronic device fabrication because the size and shape of nanostructures as well as the band structures can be modified by annealing [12,13]. This letter shows that RTA plays a major role in modifying the electronic structure and in the improvement of material quality.

## Methods

The GaAs quantum ring sample is grown on a (100) heavily doped p-type GaAs substrate by molecular beam epitaxy technique. A 0.5-μm undoped GaAs buffer layer is grown at 580°C, followed by a 30-nm Al_0.33_Ga_0.67_As barrier layer. Subsequently, As valve is fully closed while the substrate is cooled down to 400°C, and a small amount of Ga, corresponding to coverage of 10 monolayer (ML) GaAs on (100) orientation, is supplied to form Ga droplets. During Ga deposition, Si cell is opened in order to dope the nanostructures with Si equivalent to 1×10^18^ cm^−3^. The Ga droplets are then irradiated with As_4_ flux and crystallized into GaAs quantum rings at the same temperature. After quantum ring formation, a thin Al_0.33_Ga_0.67_As cap layer (10 nm) is deposited over the quantum ring at 400°C. Subsequently, the substrate temperature is raised to 600°C for the deposition of another 20 nm Al_0.33_Ga_0.67_As. The GaAs/Al_0.33_Ga_0.67_As structure is repeated six times to form the stacked multiple quantum ring structures. After the growth of multiple quantum rings, an emitter layer of 150 nm n-type GaAs with Si doped to 1×10^18^ cm^−3^ is grown. Finally, the solar cell structure is finished by a 50-nm highly Si-doped GaAs contact layer. In order to make a fair comparison in terms of effective bandgap, a quantum well solar cell used as a reference cell is fabricated with the same growth procedures, except for the quantum well region. The multiple quantum wells with GaAs coverage of 10 ML are grown, instead of the fabrication of quantum rings using droplet epitaxy. An uncapped GaAs quantum ring sample is also grown using the same procedures for atomic force microscopy (AFM) measurement. The high-resolution X-ray diffraction reciprocal space mapping (RSM) of the strain-free solar cell sample was analyzed by an X-ray diffractometer (Philips X’pert, PANalytical B.V., Almelo, The Netherlands).

Rapid thermal annealing is performed on four samples in N_2_ ambient in the temperature range of 700°C to 850°C for 2 min. The samples are sandwiched in bare GaAs wafers to prevent GaAs decomposition during high-temperature annealing. The solar cells are fabricated by standard photolithography processing. An electron beam evaporator is used to deposit Au_0.88_Ge_0.12_/Ni/Au and Au_0.9_Zn_0.1_ n-type and p-type contacts, respectively. Life-off is used to create the top grid after metal deposition. Continuous wave photoluminescence (PL) measurements are performed using the 532-nm excitation from an Nd:YAG laser with a spot diameter at the sample of 20 μm at 10 K. Two excitation power intensities of the laser are used: *I*_L_ = 0.3 W/cm^2^ and *I*_H_ = 3,000 W/cm^2^. The *J*-*V* curves of solar cells are measured under an AM 1.5G solar simulator.

## Results and discussion

**Figure 1:**
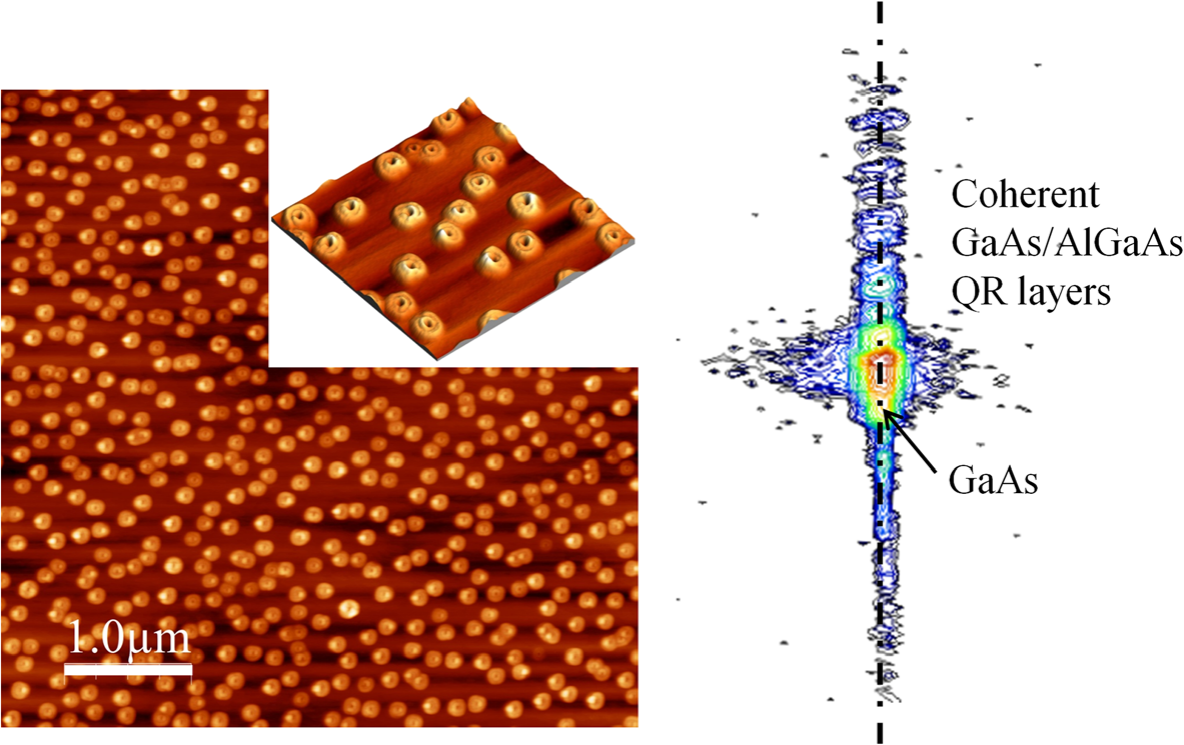
**AFM images of surface (left) and reciprocal space map of GaAs/Al**_**0.33**_**Ga**_**0.67**_**As quantum ring solar cell (right).**

The surface morphology of the uncapped GaAs/Al_0.33_Ga_0.67_As quantum ring sample is imaged by an AFM, as shown in Figure 1. The image shows quantum ring structures with a density of approximately 2.4×10^9^ cm^−2^. The inset AFM image shows double quantum rings. Figure 1 also shows the results obtained for 2D-RSM around the asymmetric 022 reciprocal lattice point (RSM 022 reflection). Strain-free quantum ring solar cell is evidenced by the RSM patterns.

**Figure 2:**
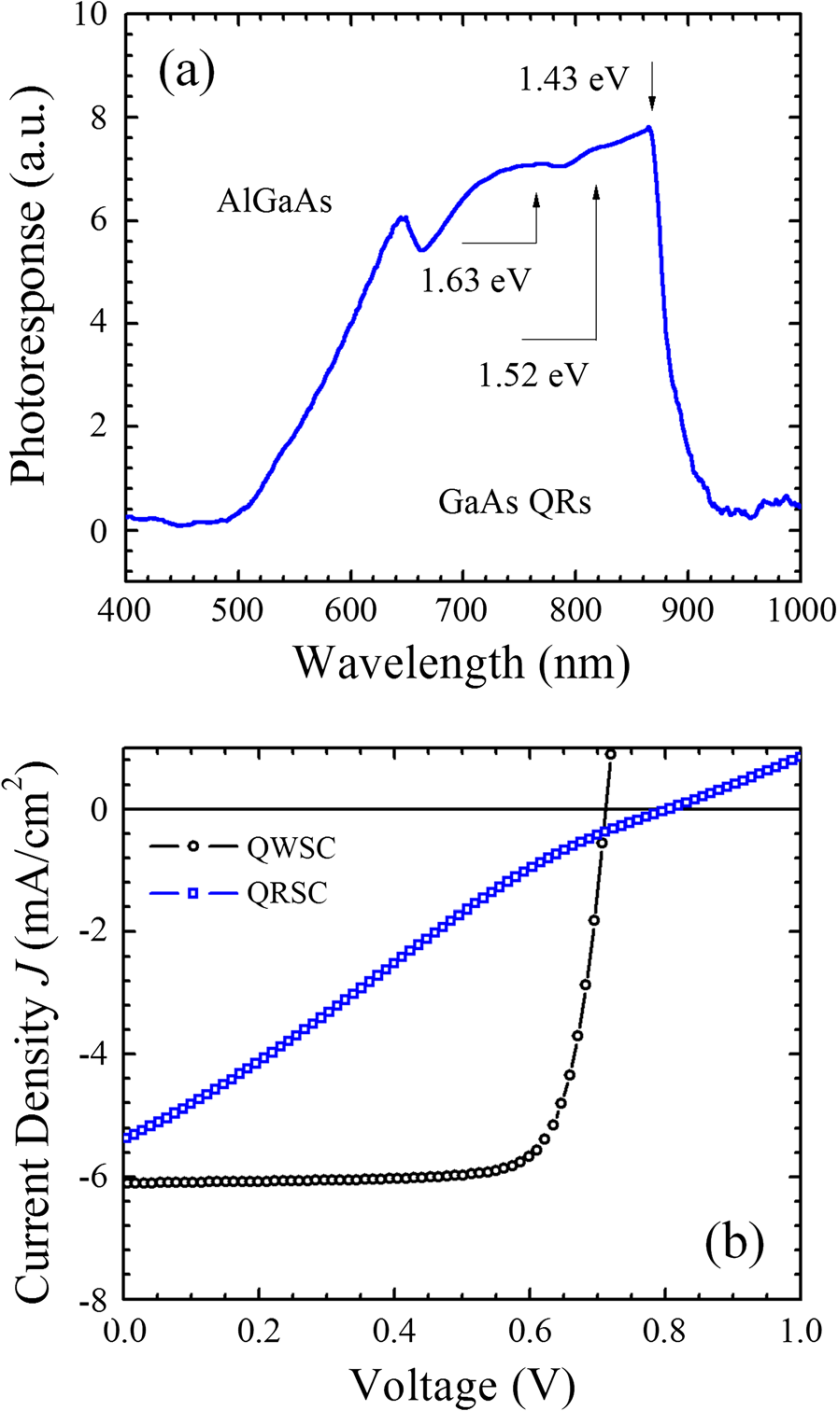
**Photoresponse of the quantum ring solar cell and current density voltage characteristics of solar cells. **(**a**) Photoresponse of the quantum ring solar cell at 300 K. (**b**) Current density voltage characteristics of a quantum ring solar cell (QRSC) and a quantum well solar cell (QWSC).

The photoresponse spectrum of the solar cell is measured using a Fourier transform infrared spectrometer interfaced with a preamplifier at 300 K without external bias voltage, as shown in Figure 2a. The spectrum shows four distinct peaks at 645, 760, 817, and 864 nm. The photoresponse peak observed around 645 nm (1.92 eV) is due to interband transitions in the Al_0.33_Ga_0.67_As barriers. The broad photoresponse band covering 760 nm (1.63 eV) and 817 nm (1.52 eV) can be assigned to the interband transitions through the energy levels in the GaAs quantum rings, while the peak around 864 nm (1.43 eV) is due to the bulk GaAs. Figure 2b shows the current density voltage characteristics of a quantum ring solar cell and a quantum well solar cell as reference cells. For the quantum ring solar cell, both the current density and fill factor are low. However, the quantum well solar cell with a similar device structure has a better performance in terms of current density and fill factor. A careful examination can reveal an increase of open-circuit voltage of the quantum ring solar cells. The IBSC is intended to increase the voltage at the expense of some of the sub-bandgap current because some of the intermediate band states are filled with electrons preventing transitions from the valence band to these filled intermediate band states [14]. Here, a plausible explanation is that the quantum ring solar cell, instead of the quantum well solar cell, forms an isolated intermediate band from the conduction band due to three-dimensional confinement and preserves the open-circuit voltage with reduced current. Moreover, since the open-circuit voltage is about the same for both quantum ring and quantum well solar cells, we also attributed the reduction in short-circuit current and fill fact of the quantum ring solar cell to the high series resistance and non-radiative recombination centers. Both quantum ring and quantum well solar cells are fabricated with similar processes, and the possibility for a difference in the contact resistance can be ruled out. Here in this study, the quantum rings and 10 nm of AlGaAs (totally 30 nm) barrier are fabricated at 400°C, which is lower than the typical growth temperature for GaAs and AlGaAs. The low-temperature growth of quantum rings and barriers is expected to generate various defects and cause degradation of material quality. These defects can act as majority carrier traps which lead to a reduction of carrier concentration and an increase in series resistance.

**Figure 3:**
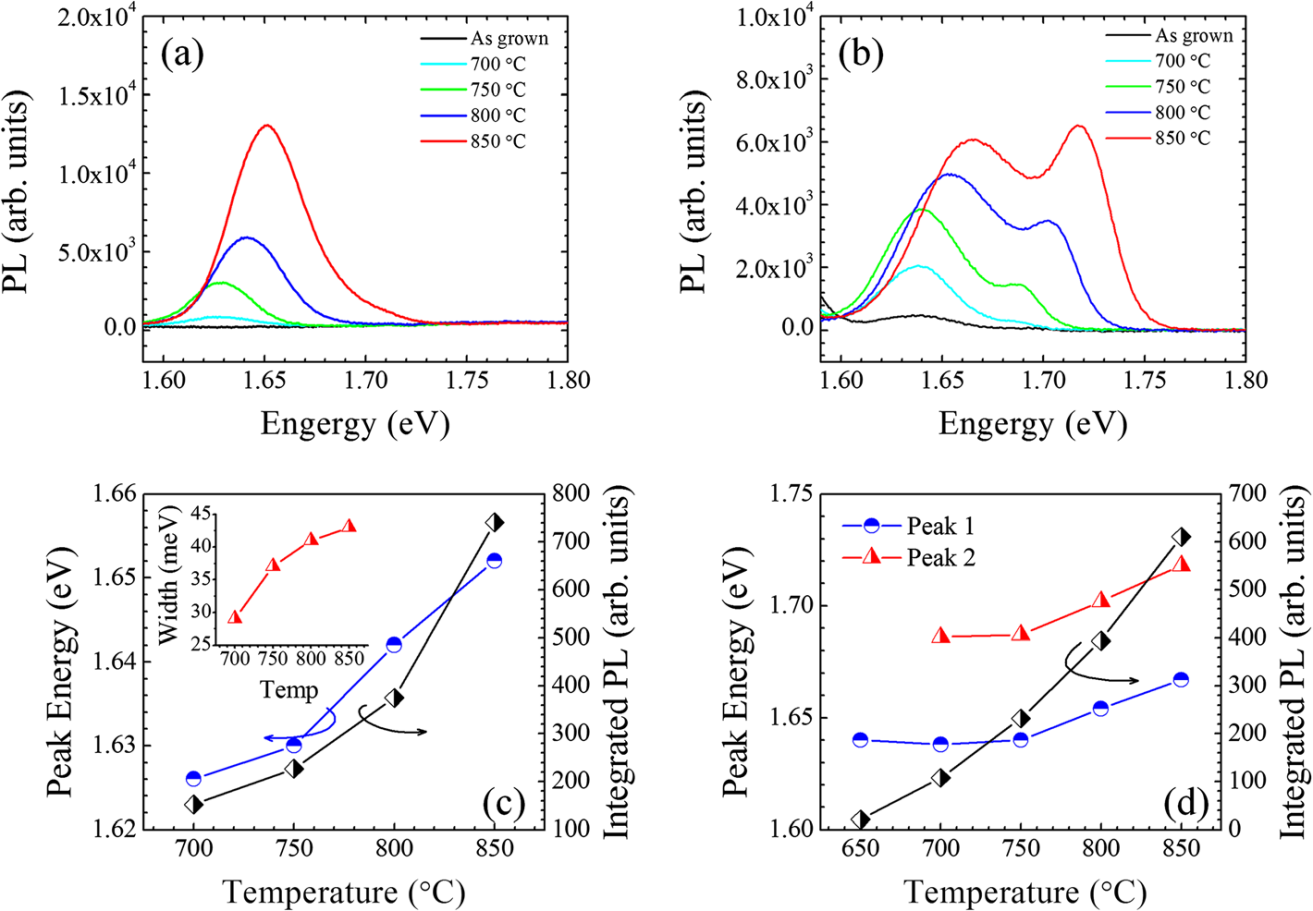
**PL spectra of solar cells and PL peak energy and integrated PL intensity. **(**a**) PL spectra of the solar cell samples annealed with different temperatures. The laser excitation power is *I*_L_ = 0.3 W/cm^2^. (**b**) PL spectra of the solar cells annealed with different temperatures. The laser excitation power is *I*_H_ = 3,000 W/cm^2^. (**c**) PL peak energy and integrated PL intensity as a function of annealing temperatures under low excitation power *I*_L_. The inset is the PL line width as a function of annealing temperatures. (**d**) PL peak energy and integrated PL intensity as a function of annealing temperatures under high excitation power *I*_H_. The data obtained from the as-grown material is plotted at 650°C.

Post-growth thermal treatments have been used to recover the material quality of quantum structures grown at low temperature. With post-growth annealing, solar cell performance has significantly improved [9]. In order to gain further insight into the properties of the quantum ring solar cells, the PL spectra of the quantum ring solar cell sample before and after rapid thermal annealing are measured and shown in Figure 3. At a laser excitation power *I*_L_ = 0.3 W/cm^2^, the PL peak at 1.64 eV appears only after post-thermal annealing and the PL spectrum intensity increases distinctly as a function of annealing temperature. This peak can be attributed to the ground energy level transition in the quantum ring, which corresponds to the photoresponse peak at 1.52 eV measured at 300 K. The PL spectra have shown a blueshift and significant broadening after thermal annealing. The integrated intensity, peak energy, and full width at half maximum of the PL spectra measured to laser excitation *I*_L_ as a function of the annealing temperature are plotted in Figure 3c. At high laser excitation *I*_H_ = 3,000 W/cm^2^, a second PL peak appears at approximately 1.7 eV after annealing, as shown in Figure 3b. The second peak is assigned to the excited state transitions in the GaAs quantum ring structures which correspond to the photoresponse peak at 1.63 eV. Similar to the quantum ring ground state transition, the PL spectra experience an emission enhancement as well as a blueshift with increasing annealing temperature (Figure 3d).

The increase in the PL yield after thermal annealing is due to the considerable improvement of material quality. Post-thermal annealing promotes the depletion of defects generated in GaAs nanostructures as well as the AlGaAs barriers processed at low temperatures. The blueshift and the broadening of the PL spectra after annealing is due to the interdiffusion of Al and Ga at the GaAs quantum ring and Al_0.33_Ga_0.67_As barrier interface. With increasing annealing temperature, the Al and Ga elements become mobilized with diffusion length as a function of annealing temperature. As a result, the concentration of Al element is increased in the GaAs quantum ring. The PL line width (PL peak 1.64 eV) changes from 29 to 43 meV as the annealing temperature increases from 700°C to 850°C (the inset in Figure 3c). The PL spectrum broadening is somehow different from the observation for InAs quantum dots. For high-temperature annealing, the size distribution and composition fluctuation of the InAs quantum dots can be improved due to the In-Ga interdiffusion [15,16]. In the case of GaAs quantum ring, the broadening of PL spectra may be explained by the gradient of Al distribution in GaAs quantum ring and barriers introduced by thermal annealing, which may be beneficial for photovoltaic applications. Compared with the In and Ga elements, the diffusion length of Al elements is short and in the range of a few nanometers due to a large Al-As bonding energy [17,18]. Therefore, a gradient of Al distribution results in the GaAs/AlGaAs interface, instead of the improvement of composition fluctuation. Additionally, the interdiffusion smooths the quantum ring and barrier interface and modifies the quantum ring geometrical shape and further electronic structures.

## Conclusions

GaAs quantum rings are fabricated by droplet epitaxy growth method. The effects of rapid thermal annealing on optical properties of quantum ring solar cells have been investigated. Thermal annealing promotes interdiffusion through depletion of vacancies and greatly enhances the material quality of quantum rings grown by low-temperature droplet epitaxy. Post-growth annealing also modifies the sharp GaAs/AlGaAs interface, and a gradient interface caused by the annealing leads to broadband optical transitions and thus improves the solar cell performance. These strain-free quantum structures with improved material quality after being treated by rapid thermal annealing may provide an alternative way to fabricate high-efficiency intermediate band solar cells. Further studies on the thermal annealing process are required to optimize quantum structures for intermediate band solar cell applications. A better correlation between morphological change and optical property enhancement during thermal annealing needs to be identified. For example, the three-dimensional quantum confinement has to be preserved while improving the optical properties after annealing.

## Abbreviations

AFM: Atomic force microscopy; IBSC: Intermediate band solar cell; PL: Photoluminescence; QD: Quantum dot; RTA: Rapid thermal annealing.

## Competing interests

The authors declare that they have no competing interests.

## Authors’ contributions

JW carried out the sample growth and device fabrication. VGD participated in the PL measurements. JW and SL carried out the XRD, AFM, J-V, and photoresponse measurements. JW, ZMW, SL, JL, and YIM participated in the statistical analysis and drafted the manuscript. JW, ZMW, ESK, and GJS conceived the study and participated in its design and coordination. All authors read and approved the final manuscript.
